# Trends in Diagnosis and Disparities in Initial Management of High-Risk Prostate Cancer in the US

**DOI:** 10.1001/jamanetworkopen.2020.14674

**Published:** 2020-08-31

**Authors:** Vishesh Agrawal, Xiaoyue Ma, Jim C. Hu, Christopher E. Barbieri, Himanshu Nagar

**Affiliations:** 1 Department of Radiation Oncology, Weill Cornell Medicine, New York, New York; 2 Division of Biostatistics and Epidemiology, Department of Healthcare Policy and Research, Weill Cornell Medicine, New York, New York; 3 Department of Urology, Weill Cornell Medicine, New York, New York

## Abstract

This retrospective study uses data from the National Cancer Database of new cancer diagnoses across the US to examine trends in proportional diagnosis rates and management of patients with high-risk prostate cancer.

## Introduction

Evidence suggests increasing rates of high-risk prostate cancer. Treatment for high-risk prostate cancer includes prostatectomy or radiotherapy. We examine trends in proportional diagnosis rates and management of patients with high-risk prostate cancer.

## Methods

The National Cancer Database (NCDB) tabulates data from more than 70% of new cancer diagnoses across the US. The NCDB was queried to identify men with high-risk prostate cancer from 2004 to 2016. Men were classified as having high-risk disease if they had clinical stage T3-T4, a prostate-specific antigen level greater than 20 ng/mL, or a Gleason score of 8-10. The eFigure in the Supplement outlines the cohort selection.

Descriptive statistics for factors were reported as frequency. The Cochran-Armitage test identified trends in treatment with time. Multivariable logistic regression examined factors associated with each treatment. All tests were 2-sided and considered significant at an α level of .05. Analyses were performed with SAS software version 9.4 (SAS Institute Inc). This study follows STROBE reporting guidelines.

## Results

Overall, 214 972 men were identified as having high-risk prostate cancer from 2004 to 2016 and 75 847 underwent prostatectomy and 104 635 underwent radiotherapy. White and black men comprised 79.2% and 16.1% of the cohort, respectively. Government-based insurance was used by 59.3% of the men. Approximately 82% of the cohort had a Charlson-Deyo comorbidity index of 0.

The proportional rates of high-risk prostate cancer increased from 11.8% to 20.4% (*P* < .001). The proportion of men undergoing prostatectomy increased from 22.8% to 40.5% (*P* < .001; Figure, A). Conversely, the rates of radiotherapy decreased from 59.7% to 43.3% (*P* < .001). External beam radiation therapy (EBRT) with a brachytherapy boost was used in 12.6% of men undergoing radiotherapy. Consistent with data presented in part A of the Figure, the odds of undergoing prostatectomy increased from 2004 to 2013 and remained consistent through 2016 (odds ratio, 2.34 [95% CI, 2.12-2.48]; *P* < .001). This trend was also observed among black men (Figure, B). The multivariable analysis appears in the Table.

**Figure.**
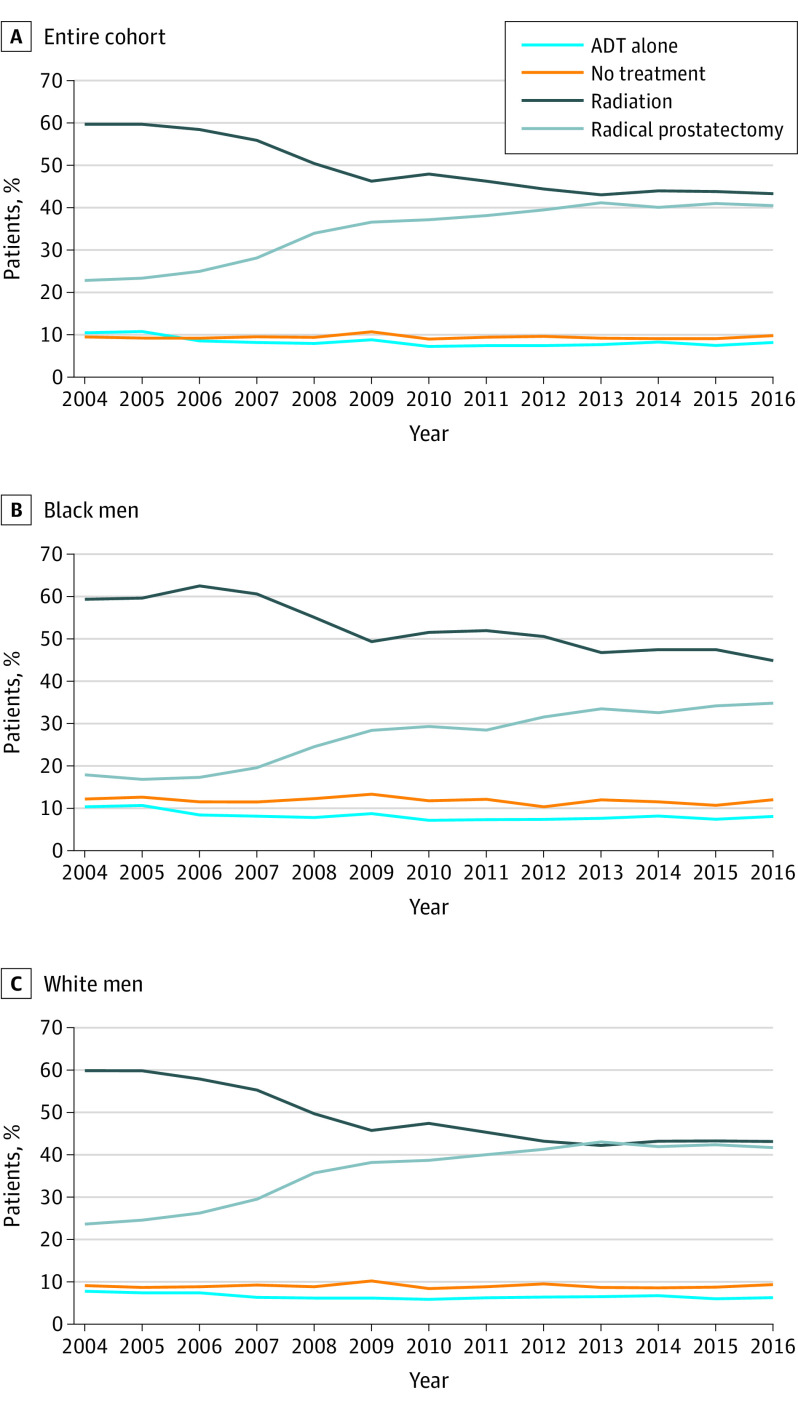
Trends in Prostate Cancer Treatment From 2004-2016 ADT indicates androgen deprivation therapy.

**Table.** Multivariable Logistic Regression for Association of Patient Characteristics With Radical Prostatectomy

	No. (%) of Patients	Odds Ratio (95% CI)	*P* value
Year of diagnosis			
2004	13 030 (6.1)	1 [Reference]	
2005	12 904 (6.0)	1.01 (0.94-1.08)	.81
2006	13 864 (6.5)	1.09 (1.02-1.17)	.01
2007	15 005 (7.0)	1.29 (1.21-1.37)	<.001
2008	16 391 (7.6)	1.70 (1.60-1.81)	<.001
2009	14 771 (6.9)	2.02 (1.89-2.15)	<.001
2010	16 972 (7.9)	2.03 (1.91-2.16)	<.001
2011	17 716 (8.2)	2.15 (2.03-2.29)	<.001
2012	16 407 (7.6)	2.32 (2.18-2.47)	<.001
2013	17 370 (8.1)	2.51 (2.36-2.67)	<.001
2014	17 919 (8.3)	2.52 (2.37-2.68)	<.001
2015	20 384 (9.5)	2.66 (2.50-2.82)	<.001
2016	22 239 (10.4)	2.72 (2.56-2.88)	<.001
Age group, y			
≤50	5638 (2.6)	1 [Reference]	
>50-60	41 690 (19.4)	0.54 (0.50-0.58)	<.001
>60-70	87 479 (40.7)	0.33 (0.31-0.35)	<.001
>70-80	63 683 (29.6)	0.08 (0.08-0.09)	<.001
>80	16 482 (7.7)	0.01 (0.01-0.01)	<.001
Gleason score			
≤6	24 016 (11.2)	1 [Reference]	
3 + 4	26 495 (12.3)	1.19 (1.13-1.24)	<.001
3 + 5	8108 (3.8)	0.96 (0.89-1.03)	.21
4 + 3	17 618 (8.2)	0.92 (0.88-0.97)	.003
4 + 4	75 260 (35.0)	0.58 (0.56-0.61)	<.001
4 + 5	45 409 (21.1)	0.64 (0.61-0.67)	<.001
5 + 4	12 686 (5.9)	0.57 (0.53-0.60)	<.001
5 + 5	5380 (2.5)	0.37 (0.34-0.41)	<.001
Prostate-specific antigen level, ng/mL			
0.1-4.0	17 694 (8.2)	1 [Reference]	
4.1-<10	75 003 (34.9)	0.91 (0.87-0.95)	<.001
10-≤20	36 245 (16.9)	0.60 (0.58-0.63)	<.001
>20	86 030 (40.0)	0.34 (0.32-0.36)	<.001
Clinical stage			
T1	115 436 (53.7)	1 [Reference]	
T2	70 356 (32.7)	0.81 (0.79-0.83)	<.001
T3	26 847 (12.5)	0.53 (0.52-0.56)	<.001
T4	2333 (1.1)	0.21 (0.18-0.24)	<.001
Charlson-Deyo comorbidity index			
0	175 464 (81.6)	1 [Reference]	
1	31 103 (14.5)	1.63 (1.58-1.68)	<.001
>1	8405 (3.9)	1.27 (1.20-1.35)	<.001
Race			
White	170 198 (79.2)	1 [Reference]	
Black	34 667 (16.1)	0.57 (0.55-0.59)	<.001
Other	10 107 (4.7)	0.96 (0.91-1.01)	.14
Geographic location			
New England	13 407 (6.2)	1 [Reference]	
Mid Atlantic	32 135 (14.9)	1.36 (1.29-1.43)	<.001
South Atlantic	45 920 (21.4)	1.13 (1.07-1.19)	<.001
Central			
East North	41 440 (19.3)	1.42 (1.35-1.50)	<.001
East South	16 174 (7.5)	2.51 (2.36-2.67)	<.001
West North	18 242 (8.5)	2.24 (2.11-2.38)	<.001
West South	13 188 (6.1)	2.70 (2.53-2.88)	<.001
Mountain	8466 (3.9)	2.04 (1.90-2.19)	<.001
Pacific	26 000 (12.1)	1.73 (1.64-1.83)	<.001
Facility type			
Community	19 183 (8.9)	1 [Reference]	
Academic	79 248 (36.9)	2.57 (2.45-2.69)	<.001
Comprehensive	89 912 (41.8)	1.72 (1.64-1.80)	<.001
Integrated	26 629 (12.4)	2.27 (2.16-2.39)	<.001
Type of insurance coverage			
Private	80 164 (37.3)	1 [Reference]	
Medicare, Medicaid, or other government	127 452 (59.3)	0.64 (0.62-0.66)	<.001
Uninsured	4148 (1.9)	0.62 (0.57-0.67)	<.001
Unknown	3208 (1.5)	0.52 (0.47-0.57)	<.001
Income quartile			
1 (lowest)	40 791 (19.0)	1 [Reference]	
2	46 970 (21.8)	1.09 (1.05-1.14)	<.001
3	50 429 (23.5)	1.11 (1.07-1.16)	<.001
4	76 782 (35.7)	1.12 (1.07-1.17)	<.001
No high school diploma, %			
<7	56 837 (26.4)	1.38 (1.32-1.44)	<.001
7-12.9	61 388 (28.6)	1.19 (1.14-1.23)	<.001
13-20.9	53 776 (25.0)	1.06 (1.02-1.09)	.003
≥21	42 971 (20.0)	1 [Reference]	
Distance, km			
≤96	193 848 (90.2)	1 [Reference]	
96-192	11 589 (5.4)	2.53 (2.40-2.67)	<.001
>192	9535 (4.4)	2.53 (2.39-2.67)	<.001
Population type			
Metropolitan	177 270 (82.5)	1 [Reference]	
Rural	4710 (2.2)	0.81 (0.75-0.88)	<.001
Urban	32 992 (15.3)	0.90 (0.87-0.94)	<.001

## Discussion

Prostatectomy rates increased from 22.8% in 2004 to 40.5% in 2016, nearly equaling radiotherapy rates by 2016. Randomized data comparing modalities do not and likely will not exist in the foreseeable future to determine optimal treatment. The ProtecT trial compared prostatectomy vs radiotherapy and showed no difference in prostate-cancer specific mortality, but did not include a significant number of patients with high-risk prostate cancer.^1^ The Prostate Advances in Comparative Evidence trial (NCT01584258) compares prostatectomy vs radiotherapy, but only includes patients with low-risk and intermediate-risk cancer.

Population-based and institutional studies report conflicting results. Boorjian et al^2^ showed improved all-cause mortality with prostatectomy compared with EBRT. Kishan et al^3^ reported improved prostate-cancer specific mortality among men with Gleason score 9-10 treated with EBRT and a brachytherapy boost vs EBRT or prostatectomy; there was no difference between EBRT and prostatectomy. Our study showed limited use of the brachytherapy boost in patients with high-risk disease.

The increase in prostatectomies may reflect increasing acceptance of population-based data suggesting superiority of prostatectomy.^2^ The increasing use of robotic approaches suggests urologists and patients may regard prostatectomies safer than previous techniques. Conversely, a decrease in radiotherapy may reflect reluctance toward recommended androgen deprivation therapy with radiotherapy.

Demographic and socioeconomic factors were associated with treatment selection for patients with high-risk prostate cancer. Black men were less likely than white men to undergo prostatectomy, which is consistent with previous studies, but our findings suggest this gap has improved over time.^4^ Men with private insurance were more likely to undergo prostatectomy. Higher income, private insurance, and treatment at an academic facility were found to be associated with use of robotic prostatectomy.^5^ Thus, the differential use of prostatectomy may reflect limited access to high-volume centers and disproportionate reimbursement for robotic techniques.

Men may prefer prostatectomy given the treatment burden of radiotherapy, which may change with shortened schedules.^6^ Prostatectomy rates have doubled since 2004 without guideline evidence suggesting its superiority. Trials are needed to guide optimal care. The findings of this study are limited by its retrospective nature.

## Notes

Supplement. **eFigure.** Flow Diagram of Patients Included for Analysis
