# Supplementary material for: Trends in Diagnosis and Disparities in Initial Management of High-Risk Prostate Cancer in the US
Source: JAMA Netw Open. 2020 Aug 31;3(8):e2014674. doi: 10.1001/jamanetworkopen.2020.14674 (PMC7489870; doi:10.1001/jamanetworkopen.2020.14674)
Supplement: Supplement. — eFigure. Flow Diagram of Patients Included for Analysis [file jamanetwopen-e2014674-s001.pdf]

Supplementary Online Content

Agrawal V, Ma X, Hu JC, Barbieri CE, Nagar H. Trends in diagnosis and disparities in initial management of high-risk prostate cancer in the US. *JAMA Netw Open*. 2020;3(8):e2014674. doi:10.1001/jamanetworkopen.2020.14674

eFigure. Flow Diagram of Patients Included for Analysis

This supplementary material has been provided by the authors to give readers additional information about their work.

© 2020 Agrawal V et al. JAMA Network Open.

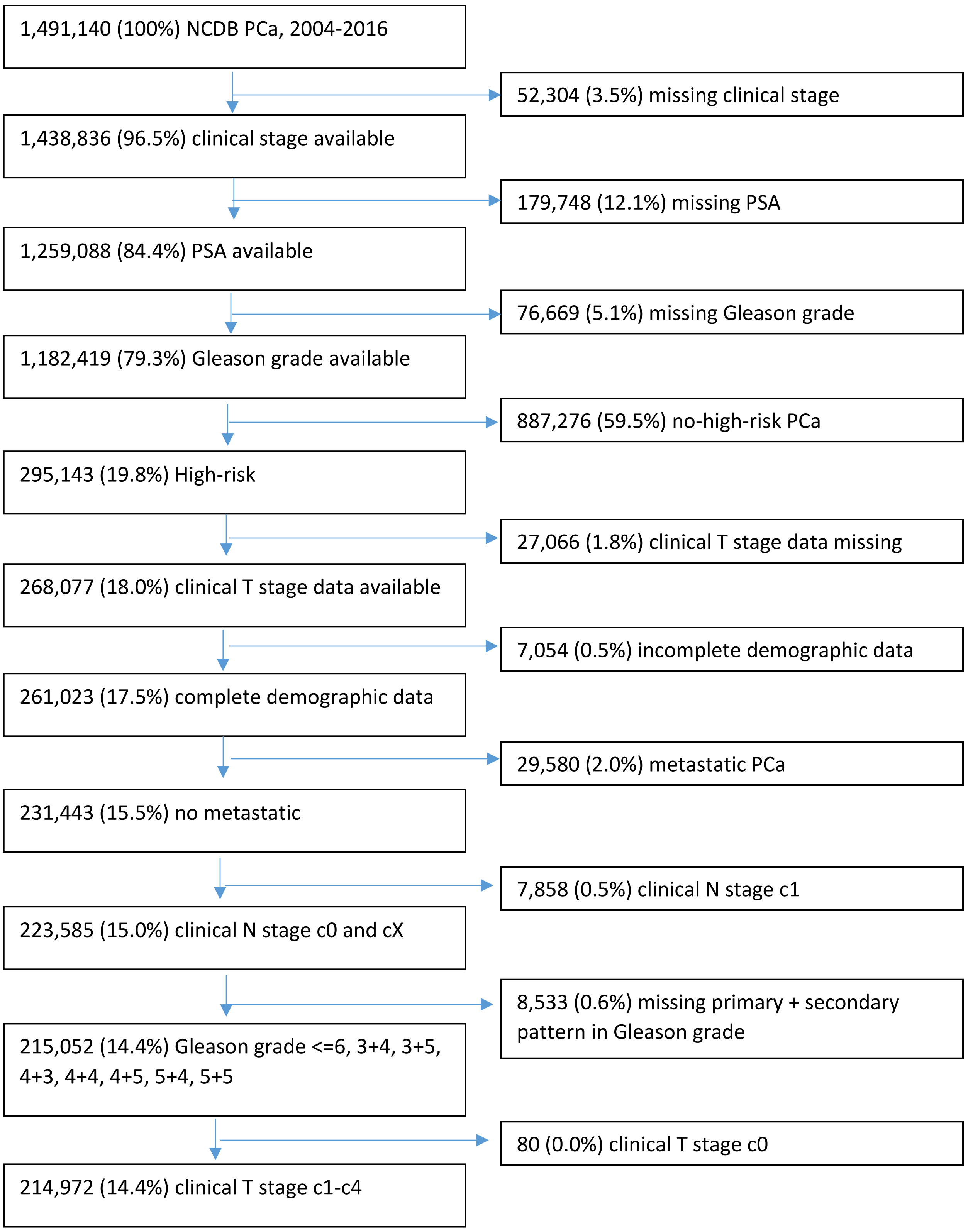

© 2020 Agrawal V et al. JAMA Network Open.
